# TolCV1 Has Multifaceted Roles During *Vibrio vulnificus* Infection

**DOI:** 10.3389/fcimb.2021.673222

**Published:** 2021-04-30

**Authors:** Yue Gong, Rui Hong Guo, Joon Haeng Rhee, Young Ran Kim

**Affiliations:** ^1^ College of Pharmacy and Research Institute of Drug Development, Chonnam National University, Gwangju, South Korea; ^2^ Clinical Vaccine R&D Center, Department of Microbiology, Combinatorial Tumor Immunotherapy MRC, Chonnam National University Medical School, Hwasun-gun, South Korea

**Keywords:** TolC, RpoS, bile salt resistance, RtxA1 secretion, *Vibrio vulnificus*

## Abstract

RtxA1 is a major cytotoxin of *Vibrio vulnificus* (*V. vulnificus*) causing fatal septicemia and necrotic wound infections. Our previous work has shown that RpoS regulates the expression and secretion of *V. vulnificus* RtxA1 toxin. This study was conducted to further investigate the potential mechanisms of RpoS on RtxA1 secretion. First, *V. vulnificus* TolCV1 and TolCV2 proteins, two *Escherichia coli* TolC homologs, were measured at various time points by Western blotting. The expression of TolCV1 was increased time-dependently, whereas that of TolCV2 was decreased. Expression of both TolCV1 and TolCV2 was significantly downregulated in an *rpoS* deletion mutation. Subsequently, we explored the roles of TolCV1 and TolCV2 in *V. vulnificus* pathogenesis. Western blot analysis showed that RtxA1 toxin was exported by TolCV1, not TolCV2, which was consistent with the cytotoxicity results. Furthermore, the expression of TolCV1 and TolCV2 was increased after treatment of the host signal bile salt and the growth of *tolCV1* mutant was totally abolished in the presence of bile salt. A *tolCV1* mutation resulted in significant reduction of *V. vulnificus* induced-virulence in mice. Taken together, TolCV1 plays key roles in RtxA1 secretion, bile salt resistance, and mice lethality of *V. vulnificus*, suggesting that TolCV1 could be an attractive target for the design of new medicines to treat *V. vulnificus* infections.

## Introduction

*Vibrio vulnificus* (*V. vulnificus*) is a halophilic Gram-negative bacterium that causes fatal primary septicemia and necrotizing wound infections, and is commonly transmitted by seawater exposure or contaminated seafood consumption (Baker-Austin et al., 2018; Park and Lee, 2018). *V. vulnificus* infections usually occur in individuals with underlying conditions such as liver diseases, diabetes, and immune disorder (Baker-Austin et al., 2018). RtxA1, a member of multifunctional autoprocessing repeats-in-toxin (MARTX) family, is a major cytotoxin of *V. vulnificus* (Kwak et al., 2011; Roig et al., 2011). Our previous studies have demonstrated that the expression of RtxA1 is dramatically increased after the close contact of *V. vulnificus* with host cells (Kim et al., 2008), and RtxA1 toxin induces the acute cell death by forming pores in the cellular membrane (Kim et al., 2013). Additionally, the expression and secretion of RtxA1 toxin are regulated by the sigma factor RpoS (Guo et al., 2018).

*V. vulnificus* TolC, an outer membrane channel protein that participates in the assembly of tripartite efflux pumps, has been reported to be involved in the secretion of RtxA1 toxin (Hwang et al., 2011). In *V. vulnificus*, there are two *Escherichia coli* (*E.coli*) TolC homologs, TolCV1 and TolCV2 (VVM0602608 and VVM0604400), showing 51.3% and 29.6% sequence identity, respectively (Lee et al., 2013). TolC is able to co-operate with several inner membrane complexes and thereby participates in the assembly of different tripartite efflux pumps, such as AcrAB-TolC (Du et al., 2014), MacAB-TolC (Du et al., 2015), EmrAB-TolC (Puértolas-Balint et al., 2020), and HlyBD-TolC (Kanonenberg et al., 2019). Some studies have demonstrated that *V. vulnificus* TolCV1 and TolCV2 can interact with *E.coli* membrane fusion protein AcrA and MacA to partially assume the efflux pump function of *E.coli* TolC (Lee et al., 2013; Lee et al., 2014b). A wide variety of substrates are directly transported across the envelope through TolC-dependent export and efflux system, which endues TolC with multiple functions and makes it to be critical for bacterial survival in the environment rich with pernicious agents or under extremal conditions (Langevin and Dunlop, 2018). Recent studies have demonstrated that AcrAB-TolC and its homologs are crucial for the drug-resistance acquisition in Gram-negative bacteria (El Meouche and Dunlop, 2018; Nolivos et al., 2019). Interestingly, *V. vulnificus* TolCV1 and TolCV2 are also associated with the efflux of diverse antibiotics (Lee et al., 2014a; Lee et al., 2015), biofilm formation (Lee et al., 2007), and iron-uptake system (Kawano et al., 2014). Several lines of evidence indicate that TolC affects virulence expression in *Vibrio cholerae*, *Francisella tularensis* and *Enterobacter cloacae* (Gil et al., 2006; Minato et al., 2011; Pérez et al., 2012) and is indispensable for bile salt resistance and colonization in *Vibrio cholerae* (Bina and Mekalanos, 2001). These fundings indicate that outer membrane TolC possesses multiple functions in various strains, which drove us to further explore more functions of TolCV1 and TolCV2 in *V. vulnificus*.

This study was conducted to further investigate the potential mechanisms of RpoS on RtxA1 secretion. First, we measured the effect of *rpoS* mutation on TolCV1 and TolCV2 expression by Western blotting. We also examined the roles of TolCV1 and TolCV2 in host factor-induced RtxA1 toxin secretion and expression, cytotoxicity to host cells, bile salt resistance and mice lethality.

## Materials and Methods

### Bacterial Strains, Plasmids and Growth Conditions

The bacterial strains and plasmids used in this study are listed in **Table 1**. Bacterial strains were reserved at −80°C in growth medium with 20% (vol/vol) glycerol. Unless stated otherwise, all *V. vulnificus* strains were propagated in Luria-Bertani broth (LB broth, Difco, Becton-Dickinson, Sparks, MD, USA) at 37°C in a shaking incubator (200 rpm).

**Table 1:** Bacterial strains and plasmids used in this study.

Bacterial strains or plasmids	Characteristics	Sources or references
***Vibrio vulnificus***		
MO6-24/O	*V. vulnificus* wild type, clinical isolate	(Reddy et al., 1992)
CMM744 (*rtxA1*-)	MO6-24/O with a deletion mutation in *rtxA1* gene	(Kim et al., 2008)
*rpoS*-	MO6-24/O with a deletion mutation in *rpoS* gene	(Guo et al., 2018)
*tolCV1*-	MO6-24/O with a deletion mutation in *tolCV1* gene	(Guo et al., 2020)
*tolCV2*-	MO6-24/O with a deletion mutation in *tolCV2* gene	This study
*tolCV1*- + pLAFR3::*tolCV1*	*tolCV1*- harbouring pLAFR3::*tolCV1*	(Guo et al., 2020)
*tolCV2*- + pLAFR3::*tolCV2*	*tolCV2*- harbouring pLAFR3::*tolCV2*	This study
		
***Escherichia coli***		
DH5α	F- *recA*1; restriction negative	Laboratory collection
SY327*λpir*	(*lac pro*) *argE*(Am) *rif nalA recA*56 *λ pir* lysogen; host for π-requiring plasmids	(Miller and Mekalanos, 1988)
SM10*λpir*	*thi thr leu tonA lacY supE recA*::RP4=-2-Tc^R^ : Mu *λ pir* lysogen, oriT of RP4, Km^R^; Conjugal donor	(Miller and Mekalanos, 1988)
		
**Plasmids**		
pLAFR3	IncP cosmid vector, Tc^R^	(Staskawicz et al., 1987)
pDM4	A suicide vector with ori *R6K sacB*, Cm^r^	(Milton et al., 1996)
pRK2013	IncP, Km^R^, Tra Rk2^+^ *repRK2 repE1*	(Ditta et al., 1980)

### Mutant Construction and Complementation

The suicide plasmid pDM4 was used to construct an in-frame *tolCV2* deletion mutant of *V. vulnificus* MO6-24/O as described previously (Guo et al., 2020). The upstream and downstream DNA fragments of *tolCV2* were amplified by PCR from *V. vulnificus* MO6-24/O chromosomal DNA (accession number NC_014966.1) (Park et al., 2011) using the primer pairs (*tolCV2*-1: 5′-GGAATTCTCTGCTGTGAGCGTTGCGCT-3′; *tolCV2*-2: 5′-GTTGCAATAATTAACCATGCGCGCCTCCCATCATC-3′) and (*tolCV2*-3: 5′-GCATGGTTAATTATTGCAACAAACATGGCAAACG-3′; *tolCV2*-4: 5′-GCTCTAGAATATCCCGTGATCACCGG-3′), respectively. These two DNA fragments were used as templates for the second crossover PCR with *tolCV2*-1 and *tolCV2*-4 as primers. The resulting PCR products were ligated into suicide plasmid pDM4 and transformed into *E. coli* SY327*λpir* and *E. coli* SM10*λpir*, generating the pDM4::△*tolCV2*, which was conjugally transferred into MO6-24/O *via* triparental mating. The stable transconjugants were selected on TCBS agar plates with chloramphenicol and then heart infusion (HI) agar plates with 10% sucrose. The mutation was verified *via* PCR and Western blot analysis.

The complementation of *tolCV2* mutant was constructed using plasmid pLAFR3 with a primer pair (*tolCV2*-F-*EcoRI*: 5′-CGGAATTCGTCCAGACATTAAAGCCG-3′; *tolCV2*-R-*PstI*: 5′-AAAACTGCAGGTTGCAATAACGCGCTC-3′). The DNA fragment containing *tolCV2* gene and flanking DNA sequence was amplified by PCR and then cloned into pLAFR3, resulting in the pLAFR3::*tolCV2*, which was introduced into *tolCV2* mutant strain by triparental mating. Stable transconjugants were selected and confirmed by PCR and Western blot analysis.

### Production of Polyclonal Anti-TolCV2 Antibody

Rabbit polyclonal anti-TolCV2 antibody was produced as described in our previous study (Guo et al., 2020). The DNA fragment encoding *tolCV2* was amplified from *V. vulnificus* MO6-24/O chromosomal DNA by PCR with the following primer pair (*tolCV2*-F-*EcoRI*: 5′-CGGAATTCATGGTTAACAAGCACCTATC-3′; *tolCV2*-R-*XhoI*: 5′-CCGCTCGAGTCATGAATGAAAAGCTCGG-3′). The resulting PCR products were then inserted into the expression vector PGEX-4T-1 (Amersham Pharmacia Biotech Inc., Piscataway, NJ) and the GST-TolCV2 fusion protein was purified by GST SpinTrap columns (GE Healthcare Life Science, Buckinghamshire, UK). The rabbit polyclonal anti-TolCV2 antibody was produced using New Zealand white rabbits, and the specificity of the polyclonal antibody against TolCV2 was confirmed by Western blotting.

### Western Blotting

Single colony of *V. vulnificus* strain was inoculated into LB broth and cultured overnight at 37°C in a shaking incubator (200 rpm). The overnight cultures were diluted 200-fold with fresh LB medium and subsequently cultured at 37°C. The pellets were washed twice with DPBS (Welgene, Gyeongsan-si, Gyeongsangbuk-do, South Korea). Bacterial cells (2×10^8^ CFU) resuspended in SDS-PAGE sample buffer were boiled at 100°C for 10 min and separated by 10% SDS-PAGE gels before being transferred to polyvinylidene fluoride (PVDF) membranes (Millipore, Bedford, MA, USA). The membranes were then blocked with 5% skim milk in Tris‐buffered saline containing 0.05% Tween 20 (TBS/T) for 2 h at room temperature and incubated with primary antibodies specific to TolCV1 (Guo et al., 2020), TolCV2 or RtxA1-D2 (Kim et al., 2013) at 4°C overnight. The membranes were rinsed with TBS/T for 1 h, incubated with horseradish peroxidase (HRP) linked anti-rabbit secondary antibody (Jackson ImmunoResearch, West Grove, PA, USA) for 1 h, and washed again with TBS/T for another 1 h. Protein bands were detected by the ECL Western blot analysis system (Advansta, Menlo Park, CA, USA). The intensity of bands was measured in arbitrary units (AU) by using ImageJ 1.50i software (National Institute of Health, USA).

### Expression and Secretion of RtxA1 Toxin in HeLa Cells Infected With *V. vulnificus* Strains

HeLa cells (Korea Cell Line Bank, Seoul, Korea) were cultured in Dulbecco’s modified Eagle’s medium (DMEM, Welgene, Daegu, Korea) containing 10% fetal bovine serum (ThermoFisher Scientific, Waltham, MA, US). *V. vulnificus* strains from cultures grown overnight in LB broth were diluted 200-fold with 10 mL of fresh LB broth in a shaking incubator at 37°C for another 3 h. HeLa cells (5×10^5^ cells/well) grown overnight in 6-well plates were washed with serum free DMEM medium before being infected with bacteria at an MOI of 20 for 120, 150, or 180 min. The supernatants (300 µL) were precipitated by the addition of 3-fold ice-cold acetone. Bacterial pellets of the supernatants and HeLa cells were lysed with the cell lysis buffer (Promega, Madison, WI, USA) containing protease inhibitor cocktail (Sigma-Aldrich, St. Louis, MO, USA) shaking on ice for 30 min and harvested by centrifugation at 13000 rpm for 10 min after scrapping. The protein concentration was quantified using the Bradford’s reagent (Bio-Rad Laboratories, USA). Equal amounts of protein were separated by NuPAGE™ 3%-8% Tris-Acetate gels (Thermo Fisher Scientific, Carlsbad, CA, USA) and subsequently subjected to Western blotting using an anti-RtxA1 antibody specific to amino acid 1492-1970 (RTX-D2 domain). Western blotting was performed as described above.

### LDH Assay

HeLa cells seeded into 48-well plates (5×10^4^ cells/well) overnight were washed with serum-free DMEM before being infected with 3 h or 9 h cultured bacterial cells of *V. vulnificus* strains at an MOI of 20 for 120, 150, or 180 min. A CytoTox96 Non-Radioactive Cytotoxicity Assay kit (Promega, Madison, WI, USA) was used to measure the amount of lactate dehydrogenase (LDH) released in the supernatants.

### The Effect of Bile Salt on TolCV1 and TolCV2 Expression

*V. vulnificus* wild-type grown overnight in LB broth were diluted 200-fold with fresh LB medium and then cultured with or without 0.02% bile salt at 37°C for 3 h or 9 h. Equal number of bacterial cells (2×10^8^ CFU) were harvested and resolved in sample buffer for Western blotting, which was performed as described above.

### Growth Determination of *V. vulnificus* Strains on TCBS Agar Plates

Thiosulphate-citrate-bile salt sucrose (TCBS) agar plate (Difco, Becton-Dickinson, Sparks, MD, USA) is usually used for the selective isolation of Vibrio species (Di Pinto et al., 2011), which contains bile salts as one of the main ingredients. To verify the growth conditions of *V. vulnificus* strains in the presence of bile salt, their overnight cultures (3 µL) were dropped on TCBS agar and then incubated at 37°C overnight.

### Measurement of Growth Rates of *V. vulnificus* Strains in the Presence of Bile Salt

Overnight cultures of *V. vulnificus* strains were diluted 200-fold with fresh LB media in the presence or absence of 0.02% bile salt. Bacterial cells were cultured in a 37°C shaking incubator and the growth was measured using a spectrophotometer at 600 nm every 2 h.

### Mice Lethality Assay

Eight-week-old female ICR mice (DBL, Umsung, South Korea) were kept under specific-pathogen-free conditions. Mice were infected with *V. vulnificus* wild-type, *tolCV1* mutant, *tolCV2* mutant, or *rtxA1* mutant strains (1×10^7^ CFU/mouse) through intraperitoneal (i.p.) injection. Five mice were tested for each group and infected mice were subsequently observed for 72 h. All procedures involving animals were performed in accordance with the guidelines of the Chonnam National University Animal Care and Use Committee (IACUC-YB-2020-81).

## Results

### Effect of *rpoS* Mutation on the Expression of Outer Membrane Proteins TolCV1 and TolCV2

**Figure 1:**
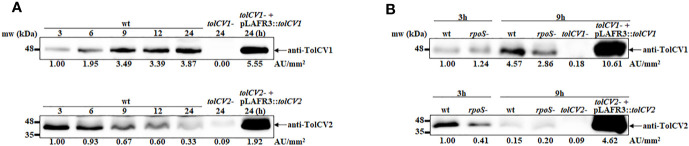
Effect of *rpoS* mutation on the expression of outer membrane proteins TolCV1 and TolCV2. Overnight cultures of each *V. vulnificus* strains were diluted 200-fold with fresh LB broth and grown in a 37°C shaking incubator. Equivalent number of bacterial cells (2×10^8^ CFU) were harvested at indicated time points followed by Western blotting with anti-TolCV1 or anti-TolCV2 primary antibodies. **(A)** Western blot analysis of *V. vulnificus* wild-type cells harvested at 3, 6, 9, 12, and 24 h cultures **(B)** Western blot analysis of *V. vulnificus* wild-type and its *rpoS* mutant cells collected at 3 h and 9 h cultures. Relative protein levels were quantified using ImageJ software. Results are representative of at least three independent experiments. Abbreviation: wt, wild-type; *tolCV1*-: *tolCV1* mutant of MO6-24/O; *tolCV1*- + pLAFR3::*tolCV1*: complementary strain of *tolCV1* mutant; *tolCV2*-: *tolCV2* mutant of MO6-24/O; *tolCV2*- + pLAFR3::*tolCV2*: complementary strain of *tolCV2* mutant; *rpoS*-: *rpoS* mutant of MO6-24/O.

We previously reported that sigma factor RpoS regulates RtxA1 expression and secretion to influence *V. vulnificus* pathogenesis (Guo et al., 2018). Hence, the current study was designed to explore whether RpoS represses RtxA1 secretion associated with outer membrane proteins TolCV1 and TolCV2. First, the TolCV1 and TolCV2 expression levels were measured at various time points and results showed that the expression levels of TolCV1 were time-dependently increased, unlike those of TolCV2, which were decreased (**Figure 1A**). Subsequently, the expression levels of TolCV1 and TolCV2 were compared between *V. vulnificus* wild-type and *rpoS* mutant strains at 3 and 9 h. Results showed that both TolCV1 and TolCV2 expression levels in the *rpoS* mutant strain were significantly lower than those in the wild-type (**Figure 1B**), indicating that RpoS acts as a positive regulator in TolCV1 and TolCV2 expressions. These data suggest that RpoS plays an essential role in the time-dependent expression of the outer membrane proteins TolCV1 and TolCV2 in *V. vulnificus*.

### Effect of *tolCV1* or *tolCV2* Mutation on Host Factor Induced-RtxA1 Expression and Secretion

**Figure 2:**
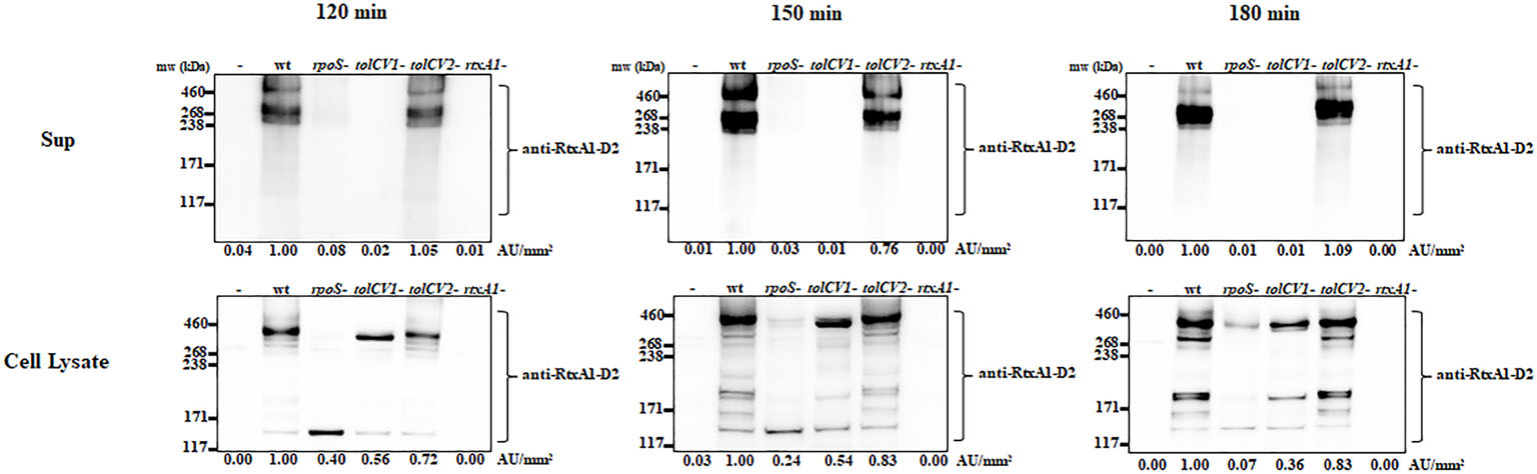
Effect of *tolCV1* or *tolCV2* mutation on RtxA1 expression and secretion after contact with host cells. HeLa cells cultured overnight in 6-well plates (5×10^5^ cells/well) were infected with either *V. vulnificus* wild-type (wt), *rpoS* mutant (*rpoS*-), *tolCV1* mutant (*tolCV1*-), *tolCV2* mutant (*tolCV2*-), or *rtxA1* mutant (*rtxA1*-) strains at an MOI of 20 for 120, 150, or 180 min. RtxA1 protein in the supernatants and cell lysates were detected by Western blot analysis with an RtxA1 antibody specific to amino acids 1492-1970 (RtxA1-D2 domain). Relative protein levels were quantified using ImageJ software. Results are representative of at least three independent experiments.

Only when the close contact of *V. vulnificus* with host cells was allowed, the expression of RtxA1 toxin would be dramatically upregulated to induce host cell death within a short time (Kim et al., 2008). To explore if TolCV1 and TolCV2 are required for the host factor induced-RtxA1 expression and secretion, the *tolCV1* and *tolCV2* mutant strains were constructed in *V. vulnificus* MO6-24/O strain. HeLa cells were infected with either *V. vulnificus* wild-type, *rpoS* mutant, *tolCV1* mutant, *tolCV2* mutant, or *rtxA1* mutant strains at an MOI of 20 for 120, 150, or 180 min. The protein levels of RtxA1 in the supernatants and HeLa cell lysates were determined by Western blotting. Similar to our previous report (Guo et al., 2018), the *rpoS* mutation resulted in a decreased level of host factor-induced RtxA1 expression in the cell lysates (**Figure 2**). RtxA1 proteins in HeLa cell lysates was detected without differences in infection with *V. vulnificus* wild-type, *tolCV1* mutant, or *tolCV2* mutant at any time (**Figure 2**). Additionally, RtxA1 protein was completely vanished in the supernatants of *tolCV1* mutant-infected HeLa cells, suggesting that RtxA1 toxin was exported by TolCV1 only, not TolCV2. Therefore, we can draw a conclusion that TolCV1 and TolCV2 do not affect the host factor-induced RtxA1 expression and TolCV1 is responsible for RtxA1 secretion.

### Effect of *tolCV1* or *tolCV2* Mutation on *V. vulnificus* Cytotoxicity to Host Cells

**Figure 3:**
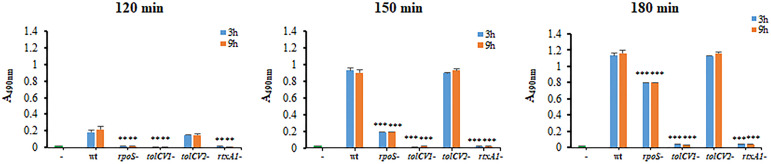
Effect of *tolCV1* or *tolCV2* mutation on *V. vulnificus* cytotoxicity to host cells. HeLa cells cultured overnight in 48-well plates (5×10^4^ cells/well) were infected with 3 h or 9 h cultured bacterial cells of *V. vulnificus* wild-type (wt), *rpoS* mutant (*rpoS*-), *tolCV1* mutant (*tolCV1*-), *tolCV2* mutant (*tolCV2*-), or *rtxA1* mutant (*rtxA1*-) strains at an MOI of 20 for 120, 150, or 180 min. The cytotoxicity of *V. vulnificus* strains was determined by measuring the amount of lactate dehydrogenase (LDH) released in the cell culture supernatants. Results are representative of at least three independent experiments. The statistical differences were analyzed by Student’s t-test (*** and ** indicate *P* < 0.001 and *P* < 0.01 versus the wild-type treated group, respectively).

To determine the roles of TolCV1 and TolCV2 in *V. vulnificus* virulence, the cytotoxicity of these strains to HeLa cells was measured by LDH assay. Consistent with Western blotting results, the *rpoS* mutant strain exhibited a decreased and delayed cytotoxicity to HeLa cells (**Figure 3**). The mutation of *tolCV1* considerably decreased cell cytotoxicity of *V. vulnificus*, but that of *tolCV2* did not exhibit a significant effect (**Figure 3**), suggesting that TolCV2 was not involved in *V. vulnificus*-induced cytotoxicity to HeLa cells. In conclusion, TolCV1 affects *V. vulnificus* cytotoxicity by controlling RtxA1 secretion, which is regulated by RpoS.

### Effect of *tolCV1* or *tolCV2* Mutation on *V. vulnificus* Resistance to Bile Salt

**Figure 4:**
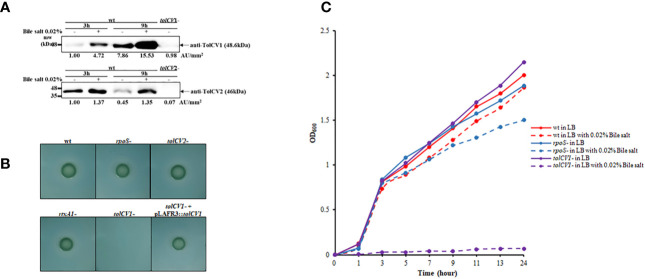
Effect of *tolCV1* or *tolCV2* mutation on *V. vulnificus* resistance to bile salt. **(A)** *V. vulnificus* cells grown overnight in LB broth were diluted 200-fold with fresh LB medium containing 0.02% bile salt for 3 or 9 h. Equivalent number of bacterial cells (2×10^8^ CFU) collected at 3 or 9 h cultures were subjected to Western blotting using anti-TolCV1 or anti-TolCV2 primary antibodies. Relative protein levels were quantified using ImageJ software. **(B)** Three microliters of overnight cultures of *V. vulnificus* strains were dropped on TCBS agar and then incubated overnight at 37°C. **(C)** Overnight cultures of *V. vulnificus* wild-type, *rpoS* mutant, or *tolCV1* mutant strains were diluted 200-fold with fresh LB media in the presence or absence of 0.02% bile salt. Bacterial cells were cultured in a shaking incubator at 37°C and the growth was measured using a spectrophotometer at 600 nm every 2 h. Results are representative of at least three independent experiments. Abbreviation: wt, wild-type; *tolCV1*-: *tolCV1* mutant of MO6-24/O; *tolCV1*- + pLAFR3::*tolCV1*: complementary strain of *tolCV1* mutant; *tolCV2*-: *tolCV2* mutant of MO6-24/O; *rpoS*-: *rpoS* mutant of MO6-24/O; *rtxA1*-: *rtxA1* mutant of MO6-24/O.

To investigate the potential roles of TolCV1 and TolCV2 after bacteria enter the human body, we measured TolCV1 and TolCV2 expression levels after treatment with the host signal bile salt. The expression of TolCV1 and TolCV2 was upregulated in LB broth with 0.02% bile salt (**Figure 4A**). Consequently, *V. vulnificus* wild-type, *rpoS* mutant, *tolCV1* mutant, *tolCV2* mutant, or *rtxA1* mutant strains were cultured on TCBS agar plates to verify their growth conditions in the presence of bile salt. The *tolCV1* mutant strain showed growth defect on TCBS agar plate, which was restored by the *in trans* complementation with a plasmid-encoded wild-type allele (**Figure 4B**). Furthermore, we monitored the growth rates of the wild-type, *rpoS* mutant, *tolCV* mutant strains in LB broth containing 0.02% bile salt. Results indicated that the growth of *tolCV1* mutant was totally abolished and that of *rpoS* mutant was slightly suppressed in LB broth with 0.02% bile salt (**Figure 4C**). Based on these results, we concluded that TolCV1 is responsible for *V. vulnificus* growth in the presence of bile salt, and is crucial for *V. vulnificus* successful infection within the host.

### Effect of *tolCV1* or *tolCV2* Mutation on Mice Lethality Caused by *V. vulnificus*

**Figure 5:**
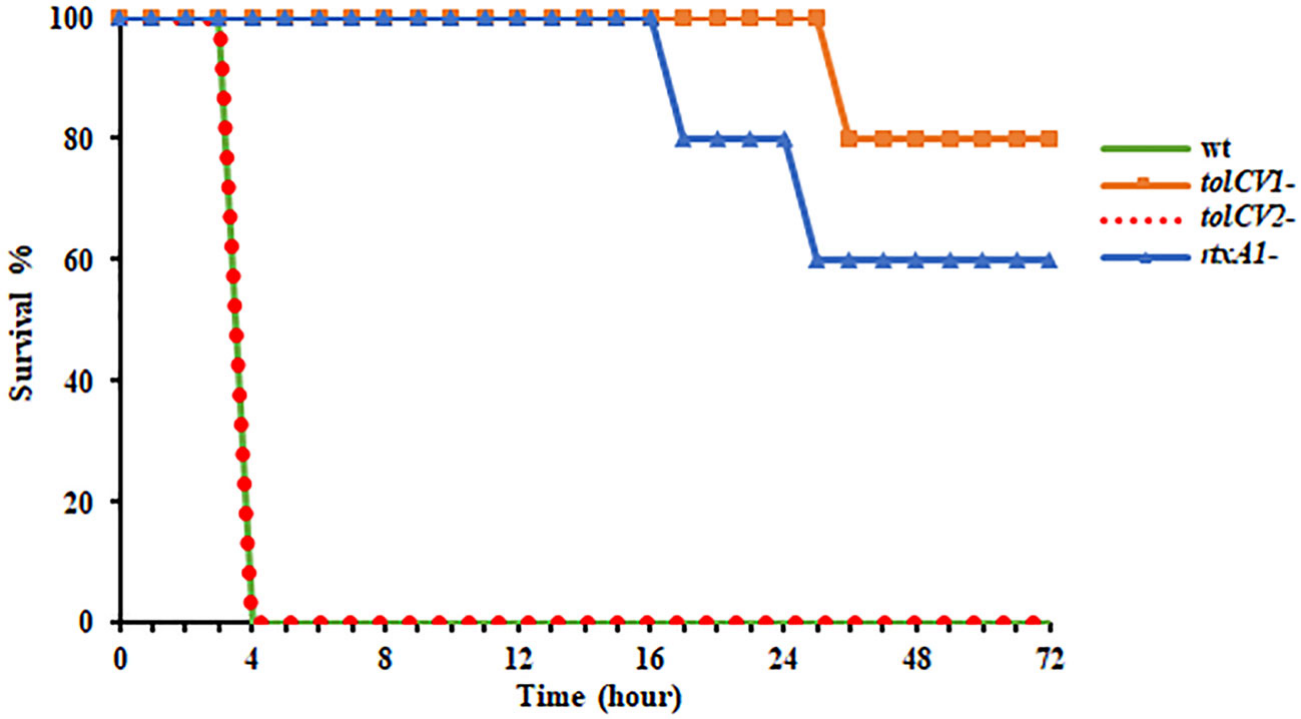
Effect of *tolCV1* or *tolCV2* mutation on mice lethality caused by *V. vulnificus*. Eight-week-old female ICR mice were infected with *V. vulnificus* wild-type (wt), *tolCV1* mutant (*tolCV1*-), *tolCV2* mutant (*tolCV2*-), or *rtxA1* mutant (*rtxA1*-) strains (1×10^7^ CFU/mouse) *via* intraperitoneal injection. Infected mice were observed for 72 h after injection and their survival times were recorded. Five mice were tested for each group.

To further study the roles of TolC proteins *in vivo*, mice were infected with either *V. vulnificus* wild-type, *tolCV1* mutant, *tolCV2* mutant, or *rtxA1* mutant strains by intraperitoneal injection and their survival times were observed in the following 72 h. As previously reported, the mice infected with *V. vulnificus* wild-type exhibited lower activity, rapid mortality, and breathing difficultly after injection, while the *rtxA1* mutant-infected mice had improved survival rates (**Figure 5**). Moreover, the mice infected with *tolCV1* mutant performed better than mice infected with *rtxA1* mutant, showing a longer survival time and higher survival rate (**Figure 5**). In contrast, the *tolCV2* mutant-infected mice were expeditiously dead and indistinguishable from the wild-type-infected mice (**Figure 5**).

## Discussion

In this study, we first found that RpoS is a positive regulatory factor of TolCV1 and TolCV2 expression (**Figure 1B**) and TolCV1 is responsible for exporting RtxA1 (**Figure 2**). Therefore, we concluded that the regulatory effect of RpoS on RtxA1 secretion was achieved by regulating the expression of TolCV1. In a previous report (Boardman and Satchell, 2004), it was suggested that RtxA1 was secreted by a Type I secretion system constituted by RtxB, RtxE, RtxD, and TolC in *Vibrio cholerae*. Therefore, RtxB, RtxE, RtxD may be the potential partners of TolCV1 collaboratively participated in the secretion of RtxA1 in *V. vulnificus*, which needs to be further confirmed. Additionally, the secretion of *V. vulnificus* hemolysin (VvhA) has been reported to be mediated by the Type II secretion system, and irrelevant with regard to TolC (Hwang et al., 2011). Subsequently, we explored the roles of TolCV1 and TolCV2 in *V. vulnificus* infection. The *tolCV1* mutation resulted in *V. vulnificus* growth defect in the presence of host signal bile salt (**Figures 4B**, **C**), which possibly explains why *tolCV1* mutation significantly reduced *V. vulnificus*-induced virulence in mice (**Figure 5**). RpoS is a stress sigma factor that is strongly induced during bacterial growth into stationary phase (Battesti et al., 2011). In the present study, we showed that TolCV1 expression time-dependently increased (**Figure 1A**), which may be caused by the accumulation of intracellular RpoS. This regulation presumably enables the bacteria to be more adaptive to stressful conditions in the stationary phase. In contrast, TolCV2 showed a time-dependently decreased expression trend (**Figure 1A**), suggesting that the expression of TolCV1 and TolCV2 was cooperatively regulated by several factors. A recently reported study revealed that the dead cells of bacterial swarms served as an “alarm signal” for live cells to increase their antibiotic resistance by releasing AcrA protein to bind with TolC located on the outer membrane of alive cells (Bhattacharyya et al., 2020). As the number of dead cells increased over time, we speculate that the gradually increased dead bacterial cells release some substances into the cultures that stimulate the expression of TolCV1 and TolCV2; however, this merits further research.

As previously reported, the outer membrane protein TolC affects virulence expression in *Vibrio cholerae*, *Francisella tularensis* and *Enterobacter cloacae* (Gil et al., 2006; Minato et al., 2011; Pérez et al., 2012). *V. vulnificus* TolCV1 and TolCV2 proteins display 78.65% and 44.44% sequence identity with *Vibrio cholerae* TolC, respectively. However, in *V. vulnificus*, the loss of outer membrane proteins TolCV1 and TolCV2 did not exhibit significant effect on the host factor-induced expression of RtxA1 toxin (**Figure 2**). Although Hwang et al. (Hwang et al., 2011) stated that TolC is responsible for RtxA1 secretion in *V. vulnificus*, whether TolCV1 or TolCV2 performed this task remained unclear. Here, we confirmed that RtxA1 toxin was exported by TolCV1 only, not TolCV2 (**Figure 2**). Besides, *tolCV1* and *tolCV2* mutant strains exhibited no difference with the wild-type strain in adhesion, and *tolCV1* mutation resulted in mild decreased motility of *V. vulnificus* in LB with 0.3% agar (data not shown).

*V. vulnificus* infections affect many people due to the intake of contaminated seafood (Chung et al., 2006). Large amounts of bile salt are distributed in the human intestinal environment, and the gastrointestinal tract is one of the most important ways for *V. vulnificus* to enter the human body. Existing evidence that TolC is indispensable for bile salt resistance and colonization in *Vibrio cholerae* (Bina and Mekalanos, 2001) encouraged us to explore the effect of TolCV1 and TolCV2 on bile salt resistance in *V. vulnificus*. The results indicated that TolCV1 is also responsible for maintaining *V. vulnificus* survival in the presence of bile salt (**Figures 4B**, **C**). Furthermore, a previous study revealed that the deletion of *rpoS* resulted in *V. vulnificus* delayed adaptation to bile salt (Chen et al., 2010), which can be explained by the downregulation of TolCV1 expression. Moreover, the mice infected with *tolCV1* mutant showed a longer survival time and higher survival rate than mice infected with *rtxA1* mutant (**Figure 5**), suggesting that TolCV1 is required for *V. vulnificus* pathogenesis and survival under *in vivo* conditions. Despite the fact that both TolCV1 and TolCV2 exhibit sequence identity in some degree to *E. coli* TolC, TolCV2 did not exhibit any comparative capacities to TolCV1. There exists an evidence that TolC is involved in the vulnibactin export of *V. vulnificus* (Kawano et al., 2014). Therefore, it is conceivable that TolCV1 and TolCV2 can also secrete other proteins or metabolites that are vital for *V. vulnificus* pathogenesis or survival. Further studies are required to comprehensively elucidate the functions of TolCV1 and TolCV2.

In the present study, we demonstrated that TolCV1 significantly influences RtxA1 secretion, bile salt resistance, and mice lethality of *V. vulnificus*. The results obtained in these experiments are encouraging, since our findings determined TolCV1 as an attractive target for developing drugs to treat *V. vulnificus* infections, which might someday lead to clinical applications.

## Data Availability Statement

Publicly available datasets were analyzed in this study. This data can be found here: https://www.ncbi.nlm.nih.gov/nuccore/NC_014966.1.

## Ethics Statement

The animal study was reviewed and approved by Chonnam National University Animal Care and Use Committee (IACUC-YB-2020-81).

## Author Contributions

YG and RG performed the experiment and analyzed the data. YG wrote the manuscript. JR contributed to the conceptual design. YK conceived and designed the study, wrote and reviewed the manuscript. All authors contributed to the article and approved the submitted version.

## Funding

This work was supported by a National Research Foundation of Korean (NRF) grant funded by the Korean government (Nos. 2018R1D1A3B07045194 and 2019R1A2C1005884).

## Conflict of Interest

The authors declare that the research was conducted in the absence of any commercial or financial relationships that could be construed as a potential conflict of interest.
